# A New Variant in the PRPF6 Gene Leading to Retinitis Pigmentosa: A Case Report

**DOI:** 10.7759/cureus.48489

**Published:** 2023-11-08

**Authors:** Armando J Ruiz-Justiz, Leonardo J Molina Thurin, Natalio Izquierdo

**Affiliations:** 1 Department of Ophthalmology, School of Medicine, Medical Sciences Campus, University of Puerto Rico, San Juan, PRI; 2 Department of Medical Education, San Juan Bautista School of Medicine, Caguas, PRI; 3 Department of Surgery, School of Medicine, Medical Sciences Campus, University of Puerto Rico, San Juan, PRI

**Keywords:** spliceosome mutation, heterozygous carrier, novel mutation, genetic variation, prpf6 gene, retinitis pigmentosa

## Abstract

We report on a case of a 34-year-old Hispanic female patient with a medical history of diabetes mellitus, thyroid disease, and cataract surgery in the right eye, who was evaluated due to progressive vision loss in both eyes. The patient had waxy pallor of the optic disc, vessel attenuation, retinal pigment epithelium (RPE) degeneration, and bony spicules OU. These findings are compatible with a diagnosis of retinitis pigmentosa (RP). Gene sequencing and deletion/duplication analysis were performed. The patient was positive for a heterozygous mutation in the *PRPF6* gene with the variant c.2228C>T (p.Thr743Ile). This *PRPF6* variant was reported as a variant of unknown significance. The Combined Annotation Dependent Depletion (CADD) score of this *PRPF6* variant is 23.5, which strengthens the idea that it could potentially be associated with RP. To our knowledge, this is the first case reported on an RP patient with the *PRPF6* variant c.2228C>T (p.Thr743Ile). Our case suggests that this *PRPF6* variant may be associated with bilateral RP. Further molecular studies are warranted to better understand the molecular changes in the *PRPF6* gene leading to RP.

## Introduction

Retinitis pigmentosa (RP) is a group of inherited retinopathies characterized by retinal dystrophy leading to progressive vision loss [1]. RP remains the most prevalent inherited retinal disorder worldwide affecting one in 3,000-8,000 patients. Its prevalence varies depending on geographic distribution [2,3].

The prevalence of RP varies widely and is primarily extrapolated from small studies conducted in various regions [4]. The frequency of RP in Europe varies from approximately one in 7,000 cases in Spain to one in 3,000-4,000 cases in Denmark and Norway [2]. In developed countries, RP affects one in 4,000 individuals, but in communities with high rates of consanguinity, it can affect up to one in 230 [4]. Griffith et al. (2022) found that there is a higher prevalence of RP in African Americans compared with the Caucasian population in South Carolina, USA [4]. A high prevalence has been reported in some Asian populations such as one in 930 in South India and one in 1,000 in China [5]. Multiple cases of RP have been reported in Latin America and the Caribbean. Yet, the global epidemiology of RP has not been documented.

Patients’ clinical symptoms begin with nyctalopia and difficult adaptation to low-light environments that later progress to significant vision loss and/or complete blindness in severe cases [6]. Symptoms may start during childhood, adolescence, or early adulthood. By the age of 40, most patients are diagnosed as legally blind. Central vision loss occurs typically by 60 years of age [2]. Since not all RP cases progress according to the same timeline, it is necessary to acknowledge that there is variation in patients' visual symptoms and RP progression.

Pathophysiology of RP is explained by progressive degeneration and apoptosis of rod photoreceptors, with subsequent involvement of cones and retinal pigment epithelium (RPE) [1,2]. These pathologic processes begin with dysfunction and loss of rods in the mid-retinal periphery where rod photoreceptors predominate. Eventually, there is cone involvement in the central retina, thus leading to central blindness. In most cases, the macula is preserved until the final stages of the disease [7].

On fundus examination, the classic signs of RP diagnosis include waxy pallor of the optic nerve, attenuation of retinal vessels, and bone spicules in the mid-peripheral retina [6]. Variations in morphology and hyperpigmentation may manifest as a result of RP. Instead of the typical bone spicules, some patients may exhibit dust-like pigmentation, while others develop nummular hyperpigmentation [6].

Retinitis pigmentosa may be classified as syndromic or non-syndromic (isolated ocular findings). Syndromic RP has been primarily associated with Usher and Bardet-Biedl syndromes [2,8]. Multiple genetic abnormalities have been identified with both forms of RP. Specifically, mutations in 80 different genes have been identified in non-syndromic RP [2]. Due to the heterogeneity of the disease, the mode of inheritance may be autosomal dominant, autosomal recessive, or X-linked, depending on the genes involved [2]. Most of the genes implicated in the pathogenesis of RP are predominantly expressed in cells of the retina or retinal pigment epithelium (RPE). However, there are mutations in constitutional genes that result in RP which are mainly responsible for the autosomal dominant inheritance pattern of the disease [3].

Besides genetic predisposition, no other risk factors for RP have been identified. However, genetic predisposition may be exacerbated by other social factors such as inbreeding due to ethnic, social, religious, emigration, and geographic isolation. RP shows no ethnic specificity, but a predominance of certain mutations has been identified in different isolated or consanguineous populations [9]. The *USH3* gene associated with type III Usher syndrome has been more frequently found in Finns and Ashkenazi Jews [9]. Sullivan et al. (2017) discovered the p.Cys147Phe mutation in the *SAG* gene among Hispanic families living in the Southwestern region of the United States [10]. In contrast with this finding, *SAG* mutations were not found in Hispanics from Cuba, Colombia, and Puerto Rico residing in the Miami area [11]. Zhang et al. (2016) reported a predominance of *PRPF31* mutation among Hispanic patients living in Miami. *EYS* is another frequently mutated gene associated with RP that has been reported in Spain and Latin America [12]. Motta et al. (2018) reported that pathogenic variants in 31 different genes lead to non-syndromic RP in Brazil. *RPGR* was found to be the most mutated gene, followed by *EYS* and *USH2A* [13]. Santos et al. (2022) identified cluster mutations in the *BBS1*, *PDE6B*, *CRB1*, *USH2A*, and *BBS7* genes in certain townships of Puerto Rico. Mutations in these genes have been identified as well in Mexican and Spanish populations [14]. Puerto Rico displays a distinct genetic makeup due to the combination of European, Indigenous, and African genes after the Spanish colonization of the island. The frequency of genetic mutations in RP varies widely among regions. 

Mutations in spliceosome genes such as *PRPF31*, *PRPF3*, *PRPF8,* and *PRPF6* have been associated with RP [15]. Models for understanding the mechanism behind spliceosome dysfunction in the pathophysiology of RP have been postulated. It has been proposed that defective transcripts or proteins accumulate due to a decrease in splicing activity in the retina or a deficiency in retina-specific mRNA maturation [16]. Deposits of these products in the retina serve as the first step in the molecular pathology of RP.

The *PRPF6* gene was identified by Tanackovic et al. (2011) [15]. It is located in chromosome 20 (20q13.33). The gene has a role in RP development with an autosomal dominant inheritance pattern [15]. The PRPF6 protein is a component of the spliceosome, an essential eukaryotic cellular structure that is required for the intron removal process in pre-mRNA molecules. The spliceosome is composed of several proteins that include five small nuclear ribonucleoproteins (snRNPs) named U1, U2, U4, U5, and U6 and numerous non-snRNPs proteins [17]. As part of the splicing process, U2 interacts with snRNPs U4 and U5 forming a tri-snRNP structure, which is mediated by PRPF6 and PRPF31 [17].

PRPF6 is considered the most important element for the stability of U5 snRNP and U4/U6 snRNP interaction [17]. Previous studies have shown that the PRPF6 protein binds to four out of the five tri-snRNPs [15]. For these reasons, PRPF6 is considered the structural core of the spliceosome and has an essential role in its assembly and disassembly. We report on an RP patient with a new variant in the *PRPF6* gene who has retinal degenerative and pigmentary changes.

## Case presentation

A 34-year-old Hispanic female patient with a medical history of diabetes mellitus, thyroid disease, and cataract surgery in the right eye complained of progressive worsening visual symptoms including night blindness during childhood. Upon physical examination, she had obesity and a body mass index (BMI) of 44.7. The patient did not have polydactyly or brachydactyly, speech, or developmental delay. The patient denied a medical history of hypogonadism, ataxia, genitourinary, or renal abnormalities. A referral for a comprehensive hearing assessment was provided to the patient. The findings of which showed that her hearing was within normal limits.

The patient underwent a comprehensive ophthalmic evaluation by at least one of the authors (NJI). The best-corrected visual acuity (BCVA) was hand motion and 20/30 in the right and left eye, respectively. Intraocular pressure (IOP) was measured to be 12 mmHg OU. Upon fundus examination, the patient had the waxy pallor of the optic disc, vessel attenuation, and bony spicules OU.

**Figure 1:**
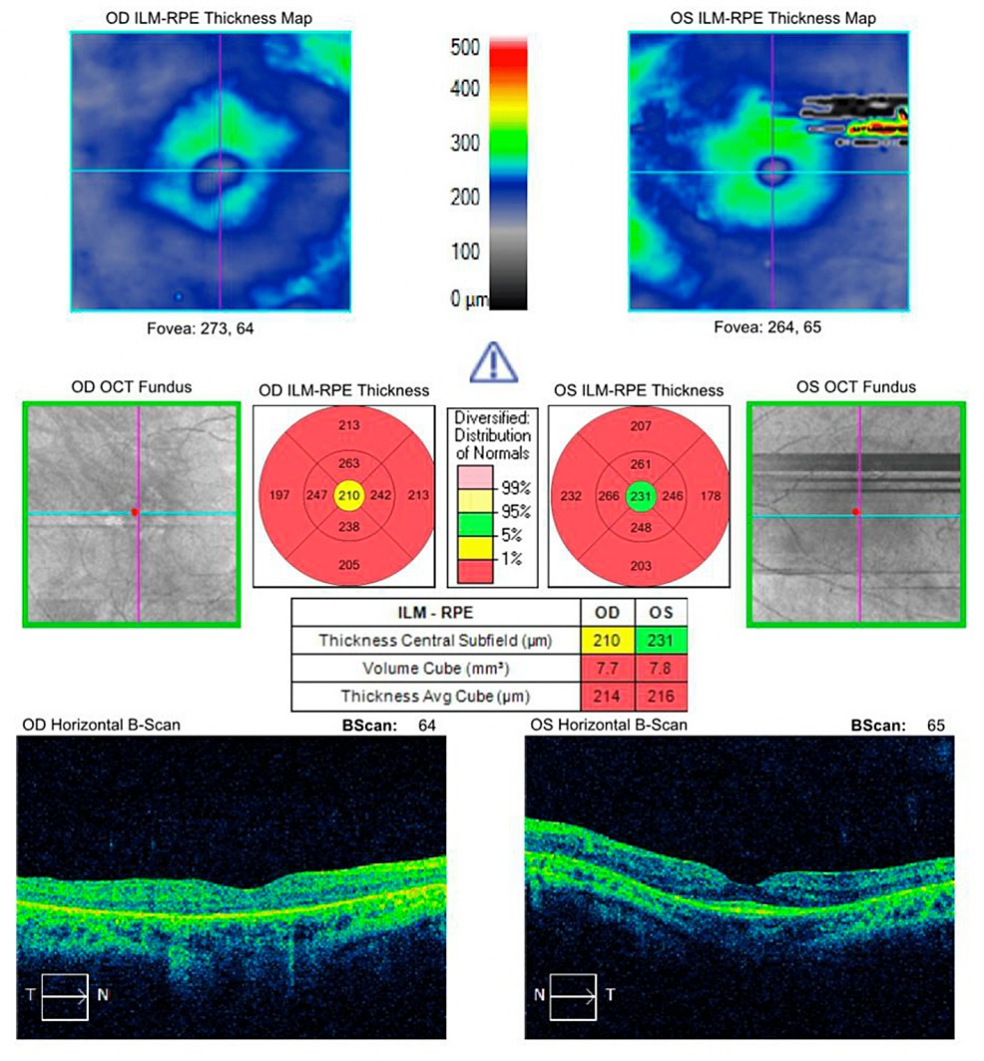
Macular optical coherence tomography (Cirrus, Zeiss) study showing a decreased macular thickness and volume. RPE: retinal pigment epithelium, OCT: optical coherence tomography, ILM: inner limiting membrane.

Upon macular optical coherence tomography (OCT) (Cirrus, Zeiss), as depicted in Figure 1, the patient had a macular thickness of 214 microns OD and 215 microns OS. The total macular volume was 7.7 mm³ OD and 7.8 mm³ OS.

**Figure 2:**
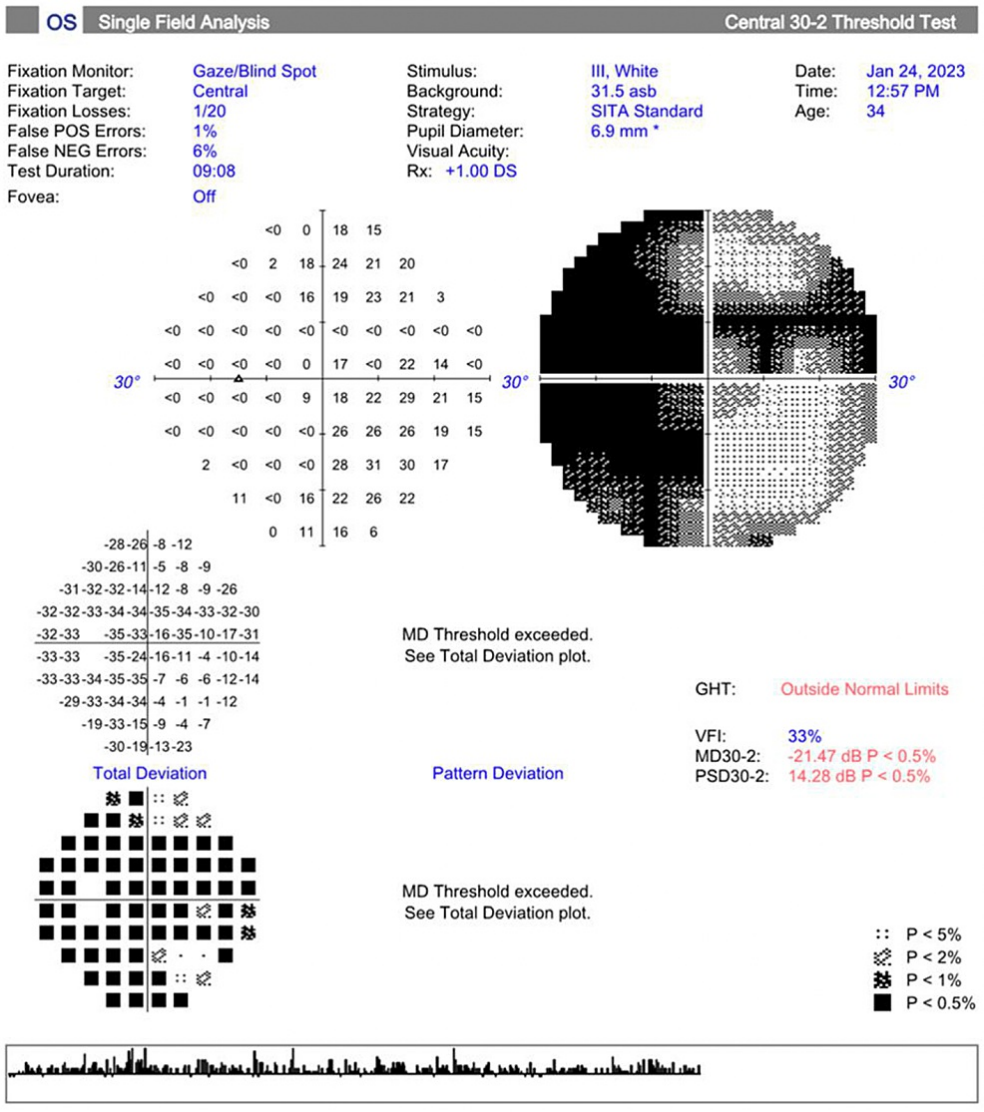
Visual field testing (30-2 Carl Zeiss Meditec, Inc.) shows a significantly decreased mean deviation (p<0.5) in the left eye. The right eye was not evaluated due to her poor visual acuity (hand motion). GHT: glaucoma hemifield test, VFI: visual field index, MD: mean deviation, PSD: pattern standard deviation.

Visual field testing (30-2 Carl Zeiss Meditec, Inc) showed a mean deviation of -21.47 dB (p<0.5) OS, as depicted in Figure 2. Due to her poor visual acuity, the right eye was not evaluated. 

Full-field electrogram (ERG) is essential for the diagnosis of RP because it assesses the electrical potential of rods and cones in photopic and scotopic stimulation. Advanced-stage patients have non-detectable ERG [6]. In our patient, ERG (LKC Technologies, Inc) showed non-recordable scotopic ERG responses OU. The photopic ERG responses were diminished, and the peak times were delayed OU. Oscillatory potentials were also diminished. These results are consistent with progressive rod-cone dystrophy.

The ocular findings and obesity made Bardet-Biedl syndrome (BBS) a differential diagnosis for the patient. The patient did not have diagnostic criteria associated with BBS. The absence of hearing loss and genes associated with Usher syndrome led to the exclusion of the syndrome.

Based on the findings of the comprehensive ophthalmic examination our patient had, a clinical diagnosis of RP was reached. A patient’s saliva sample was obtained and submitted for DNA analysis. The inherited retinal disorder (IRD) panel from the Invitae Corporation was used. Through gene sequencing and deletion/duplication analysis using next-generation sequencing (NGS), 330 genes with potential implications in RP were analyzed. It was determined to have a heterozygous mutation in the *PRPF6* gene with the variant c.2228C>T (p.Thr743Ile). This result was reported by the laboratory as a variant of uncertain significance.

## Discussion

Previous studies have reported that patients with mutations in the *PRPF6* gene have RP [15,16,18]. Our patient had symptoms such as nyctalopia and ocular findings including poor visual acuity, waxy pallor of the optic disc, vessel attenuation, and bony spicules in both eyes. Upon macular OCT, as shown in Figure 1, the patient had decreased volume and thickness bilaterally. Visual field mean deviation was significantly decreased in the left eye, as shown in Figure 2. Flash ERG response was extinguished in both eyes. All of these findings are compatible with end-stage RP.

Tanackovic et al. (2011) reported 188 families with a history of autosomal dominant RP that were screened for mutations in the 21 exons of the *PRPF6* gene on chromosome 20 [15]. They reported the c.2185C>T mutation in the exon 16, which causes an Arg>Trp substitution at codon 729 (p.Arg729Trp). This was the first mutation identified in the *PRPF6* gene associated with RP. Our patient had the variant c.2228C>T (p.Thr743Ile). The patient’s variant had not been previously associated with RP.

The PRPF6 protein is needed for adequate spliceosome function [15-17]. It has been demonstrated that mutations in the *PRPF6* gene, which lead to an aberrant spliceosome function, have a role in the molecular mechanism of the disease. Our patient’s variant c.2228C>T (p.Thr743Ile) was classified as a variant of uncertain significance (VUS) due to the lack of evidence documenting its association with RP. To our knowledge, this is the first report of a patient with RP who has the *PRPF6* variant c.2228C>T (p.Thr743Ile).

Combined Annotation Dependent Depletion (CADD) score is a method for predicting the deleteriousness of insertion, deletion, or single nucleotide variations in the human genome [19]. By comparing variants that have survived natural selection with hypothetical mutations, the CADD score combines several annotations into a single metric [19]. A significant association with allelic diversity and pathogenicity has been attributed to the CADD score. It is estimated that variants with a CADD score above 20 could be among the 1% of most deleterious mutations that might emerge in the human genome [19]. The UCSC Genome Browser (https://genome.ucsc.edu) estimates that our patient’s* PRPF6* variant c.2228C>T (p.Thr743Ile) has a CADD score of 23.5 (CADD score version 1.6) [20]. This result strengthens the idea that this *PRPF6* variant could potentially be associated with RP.

Wilkie et al. (2008) showed that missense mutations could potentially result in RP [21]. This study showed that missense mutations in the *PRPF31 *gene impair the spliceosome function in the retina, causing either premature transcription termination or protein insufficiency. These findings support the theory that splicing efficiency could be directly impacted even by missense mutations. In our patient’s *PRPF6 *variant c.2228C>T (p.Thr743Ile), the neutral and polar amino acid threonine is replaced with the neutral and non-polar amino acid isoleucine at codon 743 of exon 17. Such a molecular alteration might impair the proper spliceosome function, leading to RP. Molecular and functional analyses are required to establish this association.

Previous studies have reported that cataract surgery may improve visual acuity in RP [22]. Our patient’s vision improved from light perception to hand motion following cataract surgery in the right eye. Although cataract surgery improved vision in the right eye, retinal changes associated with RP persisted. Visual acuity remained low due to advanced retinal dystrophy. This finding further supports our hypothesis that the most substantial visual impairment in this patient was due to RP rather than cataract. This patient’s diagnosis is further supported by the presence of bilateral rod-cone degeneration shown in the ERG.

Disease due to mutations in the *PRPF6* gene has an autosomal dominant inheritance pattern [15]. Our patient was found heterozygous for the unreported variant c.2228C>T (p.Thr743Ile). Our findings suggest that this *PRPF6* variant could be a potential cause of autosomal dominant RP. Due to the implications of this mutation potentially having an autosomal dominant inheritance pattern, it is necessary to further investigate its association with RP. Further molecular studies evaluating *PRPF6* gene mutations are warranted to better understand the molecular changes that the various variants could produce in spliceosome function thus leading to RP. Further, it is possible that this mutation may occur in other Caribbean islands.

## Conclusions

Retinitis pigmentosa remains a clinical diagnosis. Although genetic testing has become an increasingly valuable instrument in the diagnosis of RP, there are still certain technological limitations to be aware of, including the occurrence of variants of unknown significance, incomplete testing, and unrecognized linked genes. The results of genetic testing in our patient showed a *PRPF6* variant that was classified as a variant of unknown significance. Although it was suggested that this mutation was unrelated to RP, the patient had symptoms and ocular findings compatible with the RP diagnosis. To our knowledge, this is the first report on a patient with bilateral RP associated with the c.2228C>T (p.Thr743Ile) variant in the *PRPF6 *gene. This case contributes to the RP literature by highlighting the need to take genetic variants, even variants of unknown significance, into account when making RP diagnoses. Our case brings attention to the continued difficulties in genetic diagnosis in this field. Further research with a larger sample size and molecular studies of this *PRPF6* variant is needed to strengthen its association with RP.
